# Superior Effects of Eccentric to Concentric Knee Extensor Resistance Training on Physical Fitness, Insulin Sensitivity and Lipid Profiles of Elderly Men

**DOI:** 10.3389/fphys.2017.00209

**Published:** 2017-04-10

**Authors:** Trevor Chung-Ching Chen, Wei-Chin Tseng, Guan-Ling Huang, Hsin-Lian Chen, Kuo-Wei Tseng, Kazunori Nosaka

**Affiliations:** ^1^Department of Physical Education, National Taiwan Normal UniversityTaipei, Taiwan; ^2^Department of Physical Education, Health and Recreation, National Chiayi UniversityChiayi, Taiwan; ^3^Department of Exercise and Health Science, University of TaipeiTaipei, Taiwan; ^4^Centre for Exercise and Sports Sciences, School of Medical and Health Sciences, Edith Cowan UniversityJoondalup, WA, Australia

**Keywords:** muscle damage, lengthening muscle contraction, senior functional fitness tests, insulin resistance, blood lipid profile

## Abstract

It has been reported that eccentric training of knee extensors is effective for improving blood insulin sensitivity and lipid profiles to a greater extent than concentric training in young women. However, it is not known whether this is also the case for elderly individuals. Thus, the present study tested the hypothesis that eccentric training of the knee extensors would improve physical function and health parameters (e.g., blood lipid profiles) of older adults better than concentric training. Healthy elderly men (60–76 years) were assigned to either eccentric training or concentric training group (*n* = 13/group), and performed 30–60 eccentric or concentric contractions of knee extensors once a week. The intensity was progressively increased over 12 weeks from 10 to 100% of maximal concentric strength for eccentric training and from 50 to 100% for concentric training. Outcome measures were taken before and 4 days after the training period. The results showed that no sings of muscle damage were observed after any sessions. Functional physical fitness (e.g., 30-s chair stand) and maximal concentric contraction strength of the knee extensors increased greater (*P* ≤ 0.05) after eccentric training than concentric training. Homeostasis model assessment, oral glucose tolerance test and whole blood glycosylated hemoglobin showed improvement of insulin sensitivity only after eccentric training (*P* ≤ 0.05). Greater (*P* ≤ 0.05) decreases in fasting triacylglycerols, total, and low-density lipoprotein cholesterols were evident after eccentric training than concentric training, and high-density lipoprotein cholesterols increased only after eccentric training. These results support the hypothesis and suggest that it is better to focus on eccentric contractions in exercise medicine.

## Introducion

Our daily physical activities and exercises consist of a combination of static (isometric), shortening (concentric), and lengthening (eccentric) muscle contractions, but eccentric contractions of the knee extensors are emphasized in some of the activities such as descending stairs or slopes and slowly sitting on a chair. One negative aspect of performing eccentric contractions is potential muscle damage characterized by delayed onset muscle soreness (DOMS) and a prolonged loss of muscle function (Clarkson et al., 1992; Howatson and van Someren, 2008; Gómez-Cabello et al., 2012; Hody et al., 2013; Jamurtas et al., 2013). Greater muscle damage is induced when eccentric contractions are performed without prior experience or with a long interval from a previous exposure to similar eccentric contractions (Howatson and van Someren, 2008; Hody et al., 2013; Hyldahl and Hubal, 2014; Ciolac and Rodrigues-da-Silva, 2016). However, it has been shown that preconditioning exercise consisting of low-intensity eccentric contractions that do not induce any symptoms of muscle damage, is effective for attenuating severe muscle damage potentially induced by maximal eccentric exercise (Chen et al., 2012, 2013). Thus, it is possible that eccentric exercise training can be prescribed without muscle damage by progressively increasing the intensity from very low to high. However, this has not been systematically investigated in previous studies, and no previous study has reported the effects of progressive resistance training on muscle damage in elderly individuals.

It has been reported that exercise training emphasizing on eccentric contractions (i.e., eccentric exercise training) increases muscle function (e.g., strength and power) and muscle mass greater when compared with concentric exercise training for young (Hortobágyi et al., 1996; Vikne et al., 2006; Paschalis et al., 2011; Elmer et al., 2012; Vogt and Hoppeler, 2014) and older individuals (Ploutz-Snyder et al., 2001; LaStayo et al., 2003; Mueller et al., 2009). For example, a classical study by Hortobágyi et al. (1996) showed that isokinetic eccentric training in which consisted of 4–6 sets of 8–12 maximal eccentric contractions of the unilateral knee extensors performed 3 sessions per week over 12 weeks increased eccentric strength 3.5 times more, concentric strength similarly and type II fiber area 10 times more, when compared with maximal isokinetic concentric training. However, this was not necessarily found in other studies (Blazevich et al., 2007; Nickols-Richardson et al., 2007; Franchi et al., 2014, 2015). For example, Franchi et al. (2015) reported that increases in maximal isometric strength and muscle mass, and muscle protein synthesis rate were similar between 4-week of eccentric and concentric training of the unilateral knee extensors when the relative intensity was matched (80% of 1RM eccentric and 80% of 1RM concentric, respectively). Thus, the superiority of eccentric to concentric exercise training is still controversial, but a recent review paper by Douglas et al. (2016) has concluded that eccentric resistance training is effective for improving muscle function to a greater extent than other modalities. It might be that eccentric exercise training does not require high-intensity contractions to induce such training effects, since relatively low-intensity eccentric contractions in eccentric cycling result in large muscle hypertrophy in elderly individuals (LaStayo et al., 2003, 2014). Gault and Willems (2013) have stated that endurance eccentric exercises characterized by high mechanical force production at a low metabolic demand (e.g., eccentric cycling, downhill walking/running) are adequate for elderly adults with or without clinical conditions, to improve their quality of life and to reduce their risk of falls. To the best of our knowledge, no previous study has compared knee extensor eccentric vs. concentric exercise training to examine whether eccentric resistance training could improve muscle and physical function of elderly individuals greater than concentric resistance training, when the intensity is progressively increased.

Eccentric exercise training has also been shown to improve insulin sensitivity and blood lipid profiles. Zeppetzauer et al. (2013) compared hiking upwards (concentric) and hiking downwards (eccentric) interventions for their effects on glucose and lipid profiles of healthy sedentary men and women, and found that the eccentric intervention resulted in significantly greater improvement of glucose tolerance and decrease in low-density lipoprotein cholesterol (LDHC) per energy expenditure when compared with the concentric intervention. Paschalis et al. (2011) reported that an 8-week eccentric resistance training but not concentric resistance training of the knee extensors performed once a week significantly improved resting insulin sensitivity and blood lipid profiles of young healthy untrained women. However, to the best of our knowledge, no previous study has compared eccentric and concentric knee extensors training for its effects on insulin sensitivity and blood lipid profiles of older adults.

Therefore, the present study investigated the effects of a 12-week progressive eccentric training in comparison to concentric training of the knee extensors on muscle function, functional physical fitness, insulin sensitivity and blood lipid profiles in elderly men. It was hypothesized that eccentric knee extensors training could be prescribed to elderly individuals without muscle damage by progressively increasing its intensity, and it would produce greater improvement of all of the outcome measures when compared with concentric knee extensors training.

## Materials and methods

### Participants

Twenty-six healthy men over 60 years of age (60–76 years) were recruited for this study. The sample size was estimated by G^*^Power (G^*^Power 3.1.9.2, Heinrich-Heine-Universität Düsseldorf, Düsseldorf, Germany; http://www.gpower.hhu.de/) based on an effect size of 0.2, an α-level of 0.05, and a power (1-β) of 0.80 for a possible difference in the increase in maximal voluntary isometric contraction torque of the knee extensors between groups. It showed that a minimum of 11 participants were necessary for each group, thus considering possible dropouts, 26 men were recruited in total.

The participants received physical examinations including blood tests at a local hospital, and were confirmed that they did not have any chronic conditions (e.g., hypertension, metabolic disorders, cardiovascular diseases), did not take any medications, and did not have any muscle, joint or bone injuries of the lower extremities. They had not performed any structured physical training program at least in the past 5 years, but they had been physically active such that they walked regularly at least three times a week for 50–60 min per day. Their mean ± SD age, height, body mass, body mass index, body fat assessed by an Inbody 3.0 Body Composition Analyzer (Biospace Co. Ltd., South Korea), maximal voluntary isokinetic (30°/s) concentric contraction torque and isometric contraction torque of the knee extensors torque were 65.9 ± 4.7 years, 164.7 ± 5.0 cm, 70.5 ± 8.0 kg, 25.9 ± 2.4 m/kg^2^, 25.5 ± 2.9%, 120.4 ± 10.3 Nm, and 151.0 ± 12.0 Nm, respectively. They were quasi-randomly allocated to either eccentric training or concentric training group (*n* = 13 per group), with an attempt to match the average baseline maximal voluntary isometric contraction torque between the groups as much as possible. No significant differences in any of the physiological characteristics shown above were observed between the groups at baseline. All participants read and signed a written informed consent form to participate in the present study, which had been approved by the Institutional Review Board Committee at the National Chiayi University. The present study was conducted in conformity with the policy statement for the use of human subjects of the Declaration of Helsinki.

The participants were requested and reminded to avoid performing any vigorous physical activities or unaccustomed exercises, maintain their normal dietary and sleep habits, and not to take any anti-inflammatory drugs (e.g., non-steroidal anti-inflammatory agent) and nutritional supplements (e.g., vitamins, protein/amino acids) for 2 weeks before and during the experimental period. They were instructed to drink sufficient water (at least 1.5 L/day) after each exercise session to avoid a potential risk of acute renal failure due to rhabdomyolysis.

### Experimental design

The participants of the eccentric training group performed only eccentric contractions of the knee extensors and those of the concentric training group performed only concentric contractions of the knee extensors with load in the 12 training sessions that were held only once a week over 12 weeks as detailed below. Thus, the independent variable of the present study was the muscle contraction mode during the resistance training: eccentric vs. concentric contractions.

The participants were familiarized with the testing procedures a week before the first exercise session. In the familiarization session, measurements of height, body mass and body fat were taken, and upper thigh circumference, and muscle soreness were assessed. The participants practiced maximal isokinetic (30°/s) concentric contractions of the knee extensors, and functional physical fitness tests often used to assess senior adults, which consisted of 30-s chair stand, 2-min step, 8-foot up-and-go, one-leg stance test with eyes-opened, 6-m tandem walk, and 6-min walk. The investigator demonstrated the eccentric exercise of the knee extensors, but no eccentric contractions were performed by the participants in the familiarization session.

All of the measurements shown above were taken again at 5 and 3 days before the first training session to establish the test-retest reliability for the baseline, and 4 days after the last training session to evaluate the effects of training from various aspects. For these time points, blood samples were also taken for the measurements of insulin sensitivity and lipid profiles after 10-h overnight fasting. The details of the blood analyses are provided in a section below. To assess muscle damage, muscle soreness was assessed before, and 1, 2, and 3 days after every training session, and maximal voluntary isokinetic concentric contraction torque and maximal voluntary isometric contraction torque were measured before, immediately after, 2 and 4 days, and plasma creatine kinase activity were measured before, and 2 and 4 days after the first and last training sessions. All of these measures were the dependent variables of the study, and changes in the variables from before to after the 12-week training, and changes in muscle damage markers before and after sessions were compared between eccentric training and concentric training groups.

### Training protocol

The participants performed a progressive eccentric training or concentric training of the knee extensors using a leg extension machine (Model: A957, Efit Sports Co. Ltd., Taiwan) once a week for 12 weeks. The knee extensors were targeted since they are one of the major muscles used in daily activities, and the exercise only requires a machine that most of exercise gyms probably have. To determine the weight to be loaded, one repetition maximal of concentric knee extension contraction (1RM) from 100° to 5° (where 0° corresponds to a knee full extended angle) was assessed with the hip joint angle being set at 70° flexion on the leg extension machine for each leg (Ben-Sira et al., 1995; Ploutz-Snyder et al., 2001; Tracy and Enoka, 2002). In the 1RM assessment, the load was progressively increased considering an estimated 1RM, with a 3-min rest between trials, and when a given load was not lifted to 15° in two successive trials, the previous load was recorded as 1RM (Ben-Sira et al., 1995; Ploutz-Snyder et al., 2001). The participants were verbally encouraged to maximally activate their knee extensors during the 1RM measurement. The 1RM was measured every week to determine the load for the week, at ~1 h prior to each training session. If the 1RM measure showed a lower value than that of the previous week, the load of the previous week was used. The number of concentric contractions in the 1RM measure was 3–5 for each leg for each training session, thus the total number of the concentric contractions over 12 weeks was 36–60 per leg. This means that eccentric training group also performed concentric contractions, but the number of concentric contractions in comparison to eccentric contractions performed in the training (540 for each leg) was small.

The load was increased from 10 to 100% of 1RM for eccentric training group, and from 50 to 100% of 1RM for concentric training group progressively over 12 weeks, and each session consisted of 3 or 6 sets of 10 eccentric or concentric contractions (Table 1). To complete an exercise session, it took 30 and 60 min for a session consisting of 3 sets and 6 sets, respectively. In eccentric training group, the participants were instructed to lower the weight from a knee extended position (10°) to a knee flexed position (100°) in 3 s, guided by the investigator who was an experienced trainer, who counted 0, 1, 2, 3 for the movement. After each eccentric contraction, the investigator returned the lever arm of the leg extension machine to the extended position, thus the participants performed concentric contraction without load (Chen et al., 2013). In concentric training group, the participants were instructed to lift the weight from a knee-flexed position (100°) to a knee-extended position (10°) in 3 s, guided by the investigator who counted 0, 1, 2, 3 for the movement. After each concentric contraction, the investigator supported the lever arm of the leg extension machine at the end of range of motion (10°) and returned the lever arm to the flexed position not to make the participants perform eccentric contraction. The interval between contractions was 10 s, and a 3-min rest between sets was inserted (Chen et al., 2013).

**Table 1:** **Intensity and volume (set x number of contractions) during the 12-week progressive concentric and eccentric training of the knee extensors (1st–12th week), and the total number of contractions (TNC) for each leg (x 2 means two legs) and total weight lifted by both legs (TWL) in the 12 weeks (mean ± ***SD*** of 13 participants)**.

**Week**	**Concentric training**		**Eccentric training**	
	**Intensity**	**Volume**	**Intensity**	**Volume**
1st	50% of 1RM	3 × 10	10% of 1RM	3 × 10
2nd	60% of 1RM	3 × 10	20% of 1RM	3 × 10
3rd	70% of 1RM	3 × 10	40% of 1RM	3 × 10
4th	70% of 1RM	6 × 10	40% of 1RM	6 × 10
5th	80% of 1RM	3 × 10	60% of 1RM	3 × 10
6th	80% of 1RM	6 × 10	60% of 1RM	6 × 10
7th	90% of 1RM	3 × 10	75% of 1RM	3 × 10
8th	90% of 1RM	6 × 10	75% of 1RM	6 × 10
9th	95% of 1RM	3 × 10	90% of 1RM	3 × 10
10th	95% of 1RM	6 × 10	90% of 1RM	6 × 10
11th	100% of 1RM	6 × 10	100% of 1RM	6 × 10
12th	100% of 1RM	6 × 10	100% of 1RM	6 × 10
TNC		540 × 2		540 × 2
TWL (kg)		34314 ± 5018		28946 ± 3428^*^

* *significant (P ≤ 0.05) difference from the concentric training group; 1RM, one repetition maximum of concentric knee extensor strength*.

A Borg's rating of perceived exertion scale with values from 6 to 20 (Borg, 1970) was used to assess subjective intensity of exercise immediately after each session. Before and during the training session, heart rate was recorded by a Polar S810 monitor (Kempele, Finland), and systolic and diastolic blood pressure were measured using an automated sphygmomanometer (MS 150f, Rossmax International Ltd., Taipei, TAIWAN) before and immediately after each training session.

### Muscle damage markers

#### Maximal concentric and isometric contraction torque

Maximal voluntary isokinetic concentric contraction torque of the non-dominant (non-kicking) leg was measured by a Biodex isokinetic dynamometer (Biodex System 3 Pro; Biodex Medical Systems, Shirley, NY, USA), in the seated position with the hip joint angle of 70° flexion, at the angular velocity of 30°·s^−1^ for the range of motion of 110° for the knee extensors (5–115°) and flexors (115–5°) for 3 continuous contractions for both directions alternatively (Chen et al., 2013, 2015; Wang et al., 2014). Maximal voluntary isometric contraction torque of the knee extensors of the non-dominant leg was also measured at the knee joint angle of 80° and the hip joint angle of 70° flexion on the isokinetic dynamometer. Each participant was asked to extend the knee joint maximally for 3 s, and this was repeated three times with a 45-s rest between attempts. The peak torque of maximal voluntary concentric and isometric contraction torque, respectively, was assessed by the software program of the Biodex Medical Systems. The highest value of the three trials for the knee extensors and flexors of maximal voluntary isokinetic concentric contraction torque, respectively, and for maximal voluntary isometric contraction torque of the knee extensors was used for further analysis.

#### Muscle soreness

Muscle soreness was assessed using a visual analog scale with a 100-mm continuous line with “not sore at all” on one end (0 mm) and “very, very sore” on the other end (100 mm). Each participant was asked to rate his perceived soreness on the visual analog scale immediately after self-stretching, using his hand to hold the instep of the foot to maximally stretch the knee extensors of the non-dominant leg while standing with the dominant leg (Chen et al., 2013; Huang et al., 2015).

#### Plasma creatine kinase activity

A 3-ml venous blood sample was collected to a vacutainer tube contained ethylenediaminetetraacetic acid (Becton, Dickinson UK Ltd., Plymouth, PL6 7BP, UK) by venipuncture from the cubital fossa region of the arm. The blood was centrifuged for 10 min to obtain plasma, and the samples were stored at −80°C until analyses of creatine kinase activity. Plasma creatine kinase activity was determined spectrophotometrically by an automated clinical chemistry analyzer (Model 7,080, Hitachi, Co. Ltd., Tokyo, Japan) using a commercial test kit (Sigma Diagnostics, St. Louis, MO, USA). All samples were analyzed in duplicate, and the mean of the two measures was used for statistical analysis. The normal reference ranges of men for creatine kinase were 38-174 IU·L^−1^.

### Assessments of training effects

#### Muscle function and upper thigh circumference

The protocols for the measurements of 1RM, maximal voluntary isokinetic concentric and isometric contraction torque explained above were also used to assess the training effects on muscle function. As for upper thigh circumference, each participant stood with his feet being ~10 cm apart and body weight being evenly distributed between feet, and the investigator took the girth measure at the mid-point between greater trochanter and epicondyle of femur of the exercised leg using a Gulick tape (Hawes and Martin, 2001). The measurements were taken three times for each time point, and the average of the three measures was used for further analysis.

#### Functional physical fitness tests

Participants had 10-min of general warm-up consisting of static and dynamic stretching exercises. Several functional physical fitness tests for senior adults were selected for the present study, which consisted of 30-s chair stand, 8-foot up-and-go, one-leg stance test with eyes-opened, 6-meter tandem walk, 2-min step and 6-min walk. Each test was performed twice except for the 6-min walk test that had one trial, and for the tests with two trials, the better score of the two was used for further analysis. The details of 30-s chair stand, 8-foot up-and-go, 2-min step and 6-min walk can be found elsewhere (e.g., Rikli and Jones, 1999; Fang et al., 2015; Lee et al., 2015), and one-leg stance test with eyes-opened and 6-m tandem walk test are described in other article (Nelson et al., 2004). The participants performed the tests in the following order with a sufficient rest (3–10 min) between tests; 30-s chair stand, 2-min step, 8-foot up-and-go, one-leg stance test with eyes-opened, 6-meter tandem walk and 6-min walk.

#### Insulin sensitivity and lipid profiles

A 5-ml venous blood was collected by a standard venipuncture to a vacutainer tube (Becton, Dickinson UK Ltd., Plymouth, PL6 7BP, UK), and centrifuged for 10 min to obtain serum, and another 3-ml of blood was collected for whole-blood glycosylated hemoglobin (HbAlC) measure taken soon after the blood sampling. Insulin sensitivity markers included resting serum glucose and insulin concentrations, homeostasis model assessment (HOMA), HbAlC and oral glucose tolerance test (OGTT). Serum glucose concentration was assayed by a Beckman Unicel DxC 600/800 Chemistry Analyzer (Beckman Coulter Unicel DxC 600/800 SYNCHRON Clinical System, Beckman Coulter Inc., Fullerton CA, USA) using a commercially available kit (GLUCm; Beckman Coulter Ireland Inc., Galway, Ireland). Serum insulin concentration was analyzed by an immunoradiometric assay kit (INS-IRMA kit; Biosource, Nivelles, Belgium) using a gamma counter system (MIC Group, Inc., MN, USA). HOMA was calculated as fasting insulin (μU·mL^−1^) x fasting glucose (mmol·L^−1^)/22.5 (Paschalis et al., 2011). HbA1C was measured by a Tosoh Automated Glycohemoglobin Analyzer HLC-723G7 (Tosho Corp. Scientific Instruments, Yamaguchi, Japan). The normal reference ranges for glucose, insulin and HbAlC was 3.9–5.6 mmol·L^−1^, 28.6–114.3 pmol·L^−1^, and 4–6%, respectively, based on the manufacturer's information. In the OGTT, after the baseline blood sample was taken as described above, each participant had a 75-g standard glucose drink, and blood samples were taken at 30, 60, 90, and 120 min after the ingestion (Green et al., 2010). Serum glucose concentration was measured as explained above, and the area under the curve from the baseline to 30, 60, 90, and 120 min was calculated (Green et al., 2010).

Blood lipid profiles consisted of serum triacylglycerols (TG), total cholesterol (TC), high-density (HDLC) and low-density lipoprotein cholesterol (LDLC). These measures were performed by a Beckman Unicel DxC 600/800 Chemistry Analyzer using commercial kits (Beckman Coulter, Inc., Fullerton, CA, USA). The reference ranges of TG, TC, HDLC, and LDLC was <1.6 mmol·L^−1^, <5.2 mmol·L^−1^, ≥1.6 mmol·L^−1^, <2.6 mmol·L^−1^, respectively, based on the manufacturer's information.

### Test-retest reliability of the dependent variable measures

Based on the two baseline measurements taken at 5 and 3 days before the first training session, the test-retest reliability of each measure was determined by coefficient of variation. The coefficient of variation for 1RM, maximal voluntary concentric contraction torque, maximal voluntary isometric contraction torque, range of motion, upper thigh circumference, muscle soreness, plasma CK activity, 6 functional physical fitness test items, glucose, insulin, HOMA, HbA1C, OGTT, TG, TC, HDLC and LDLC ranged between 0% (muscle soreness) to 24.8% (one-leg stance test with eyes-opened), and most of them were under 10%. The variables that exceeded 10% in coefficient of variation were many of the functional physical fitness tests; 30-s chair stand (14.6%), one-leg stance test with eyes-opened (24.8%), and 6-m tandem walk (14.0%).

### Statistical analyzes

Data were assessed by a Shapiro–Wilk test for the normality and a Levene test for the homogeneity of variance assumption. These tests showed that the data of all dependent variables were normally distributed, and the variance was assumed homogenous. Baseline values of each variable were compared between eccentric training and concentric training groups by a *t*-test. A *t*-test was used to compare between eccentric training and concentric training groups for the total weight lifted, and heart rate, rating of perceived exertion, systolic, and diastolic blood pressure immediately after training for each session. Changes in the dependent variables from the baseline to post-training were compared between the groups by a mixed-design two-way analysis of variance (ANOVA) for raw data, and their normalized changes were compared between groups by a *t*-test. Changes in muscle damage markers before and after the first and the last training sessions were compared between groups by a mixed-design two-way ANOVA. When the ANOVA showed a significant interaction (group × time) effect, a Tukey's *post-hoc* test was performed to compare between groups for each time point. Statistical significance was set at *P* ≤ 0.05. Data were presented as mean ± *SD*, unless otherwise stated.

## Results

### Baseline measurements

Baseline values of the dependent variables are shown in Table 2. No significant differences (*P* > 0.05) in any of the variables were found between the eccentric training and concentric training groups.

**Table 2:** **Average (± ***SD***) values of muscle function (Muscle) and functional physical fitness (FPF) as well as blood (insulin sensitivity and lipid profiles) parameters before the 12-week training of the knee extensors for eccentric (ET) and concentric training (CT) groups**.

**Muscle & FPF parameters**	**Groups**	**Mean ±*SD***	**Blood parameters**	**Groups**	**Mean ± SD**
1RM (kg)	ET	29.8 ± 2.9	Glucose (mmol/L)	ET	5.52 ± 0.19
	CT	30.5 ± 3.1		CT	5.47 ± 0.16
MVCcon (Nm)	ET	119.8 ± 10.3	Insulin (pmol/L)	ET	64.80 ± 6.79
	CT	121.1 ± 10.7		CT	66.94 ± 6.70
MVCiso (Nm)	ET	149.2 ± 11.7	HOMA (A.U.)	ET	2.29 ± 0.26
	CT	152.7 ± 12.6		CT	2.27 ± 0.25
CIR (mm)	ET	497.3 ± 20.4	HbA1C (%)	ET	5.36 ± 0.08
	CT	499.9 ± 22.9		CT	5.55 ± 0.10
CS (times)	ET	17.1 ± 2.7	OGTT (mmol/L/2 h)	ET	39.18 ± 2.90
	CT	16.0 ± 2.0		CT	38.70 ± 2.96
2MS (times)	ET	81.2 ± 6.6	TG (mmol/L)	ET	1.60 ± 0.15
	CT	83.4 ± 6.1		CT	1.56 ± 0.13
8UG (s)	ET	7.2 ± 0.4	TC (mmol/L)	ET	5.08 ± 0.14
	CT	7.3 ± 0.4		CT	5.13 ± 0.13
OLST (s)	ET	38.9 ± 9.3	HDLC (mmol/L)	ET	1.21 ± 0.06
	CT	41.1 ± 10.8		CT	1.24 ± 0.05
TW (s)	ET	29.6 ± 4.5	LDLC (mmol/L)	ET	2.94 ± 0.07
	CT	29.2 ± 3.8		CT	3.00 ± 0.09
6 MW (m)	ET	515.8 ± 35.5			
	CT	510.4 ± 38.3			

*Muscle and FPF parameters consist of one repetition maximum of concentric knee extensor strength (1RM), maximal voluntary concentric (MVCcon) and isometric contraction torque of the knee extensors (MVCiso), upper thigh circumference (CIR), 30-s chair stand (CS), 2-m step (2MS), 8-foot up-and-go (8UG), one-leg stand with eyes open (OLST), 6-meter tandem walk (TW), and 6-m walk (6MW). Blood parameters includes glucose, insulin, fasting homeostasis model assessment (HOMA), whole blood glycosylated hemoglobin (HbA1C), glucose area under the curve for 2-h in oral glucose tolerance test (OGTT), triacylglycerols (TG), total cholesterol (TC), high-density lipoprotein cholesterols (HDLC) and low-density lipoprotein cholesterols (LDLC)*.

### Training

All participants completed all 12 training sessions as instructed. In the 11th and 12th weeks, the load was 100% of 1RM maximal knee extension concentric contraction of for both groups, but most participants were able to complete the exercise without any assistance and some required minimum assistance for weaker angles. Both eccentric training and concentric training groups completed the same total number of contractions (540 contractions of each leg, 1,080 contractions in total) over the 12-week training period (Table 1). The amount of total weight lifted during the 12-week period was significantly greater for concentric training group (34,314 ± 5,018 kg) than eccentric training group (28,946 ± 3,428 kg).

Heart rate measured during eccentric training (e.g., 1st: 73.5 ± 5.4 beats/min, 12th: 76.9 ± 5.6 beats/min) and concentric training (1st: 79.5 ± 5.7 beats/min, 12th: 81.2 ± 6.4 beats/min) was not significantly different from the baseline values for both groups, and no significant difference between eccentric training and concentric training groups were evident. With increasing the intensity and volume of exercise over 12 sessions, RPE and systolic blood pressure measured after each session increased (*P* ≤ 0.05). Rating of perceived exertion after the 1st and 12th sessions was 9.7 ± 1.8 and 10.3 ± 3.0, respectively for concentric training group, and 7.7 ± 1.4 and 8.1 ± 1.1, respectively for eccentric training group. Systolic blood pressure after the 1st and 12th sessions was 120.6 ± 6.0 mmHg and 127.6 ± 8.7 mmHg, respectively for concentric training group and 116.5 ± 5.4 mmHg and 119.2 ± 7.3 mmHg, respectively for eccentric training group. For all sessions, post-session rating of perceived exertion and systolic blood pressure were greater (*P* ≤ 0.05) for concentric training group than eccentric training group. No significant changes in diastolic blood pressure (around 70 mmHg in average) were seen from pre- to post-session both groups.

### Muscle damage

The first eccentric training and concentric training sessions did not result in significant changes in maximal voluntary concentric contraction torque, maximal voluntary isometric contraction torque, visual analog scale of muscle soreness, plasma creatine kinase activity. After the 12th session, maximal voluntary concentric contraction torque decreased (*P* ≤ 0.05) immediately after exercise, but returned to the baseline by 2 days post-exercise. No significant changes in plasma creatine kinase activity were evident after the 12th session for both groups. No muscle soreness was developed after any training sessions for both groups. When comparing eccentric training and concentric training groups for changes in muscle damage markers after the 1st and 12th sessions, no significant differences were evident.

### Effect of training

#### Muscle function, upper thigh circumference and functional physical fitness

**Figure 1:**
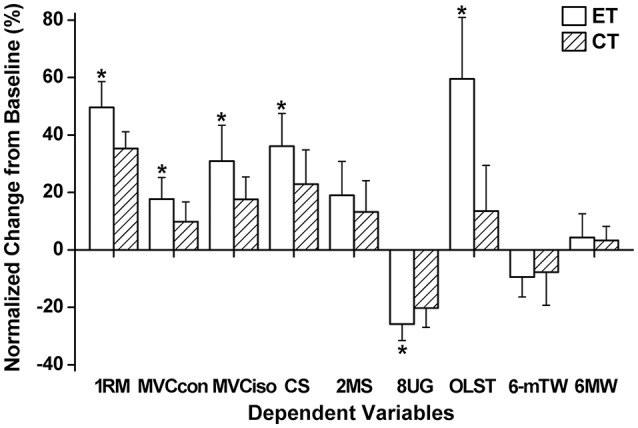
**Normalized changes (mean ± ***SD***) in one repetition maximum of concentric knee extensor strength (1RM), maximal voluntary concentric (MVCcon) and isometric contraction torque of the knee extensors (MVCiso), 30-s chair stand (CS), 2-m step (2MS), 8-foot up-and-go (8UG), one-leg stand with eyes open (OLST), 6-meter tandem walk (6-mTW), and 6-m walk (6MW) from baseline (0%) to post-training measures for eccentric (ET) and concentric training groups (CT)**. ^*^A significant (*P* < 0.05) difference from CT group.

Significantly greater increases in maximal voluntary concentric contraction torque (18 vs. 10%), maximal voluntary isometric contraction torque (31 vs. 18%), 1RM (49 vs. 35%), and upper thigh circumference (1.1 vs. 0.5%) were evident after 12-week training for eccentric training group than concentric training group (Figure 1).

Following 12-week eccentric training and concentric training, significant (*P* ≤ 0.05) improvement of all functional physical fitness tests were evident from the baseline (Figure 1). The extent of the improvement of 30-s chair stand (eccentric training group: 36%, concentric training group: 23%), 8-foot up-and-go (eccentric training group: −26%, concentric training group: −21%) and one-leg stance test with eyes-opened (eccentric training group: 59%, concentric training group: 13%) was significantly greater (*P* < 0.05) for eccentric training group than concentric training group, but no significant differences between eccentric training and concentric training groups were found for other tests.

#### Insulin sensitivity and lipid profile

**Figure 2:**
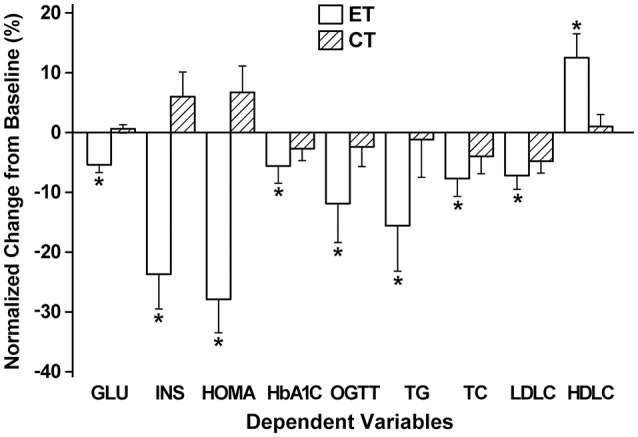
**Normalized changes (mean ± ***SD***) in fasting glucose (GLU), insulin (INS), homeostasis model assessment (HOMA), whole blood glycosylated hemoglobin (HbAlC), 2-h oral glucose tolerance test (OGTT), triacylglycerols (TG), total cholesterol (TC), low- (LDLC) and high-density lipoprotein cholesterols (HDLC) from baseline (0%) to post-training measures for eccentric (ET) and concentric training groups (CT)**. ^*^A significant (*P* < 0.05) difference from CT group.

Insulin sensitivity improved significantly after eccentric training (resting glucose: −5%, insulin: −24%, HOMA: −28%, HbA1C: −6%, OGTT: −12%), but this was not the case for concentric training (Figure 2). All blood lipid profile markers improved after eccentric training (TG: −16%, TC: −8%, HDLC: 12%, LDLC: −7%), but this was limited to LDLC after concentric training, and the magnitude of the improvement of all variables was significantly greater for eccentric training group than concentric training group (Figure 2).

## Discussion

This study tested the two hypotheses; (1) eccentric training of the knee extensors could be prescribed to elderly individuals without muscle damage by progressively increasing its intensity and volume, and (2) the eccentric training would result in greater improvement of all parameters associated with fitness and health when compared with concentric training. The results showed that (1) no DOMS was developed after any sessions and no significant changes in blood markers of muscle damage were found after the first and last training sessions, and changes in maximal voluntary concentric contraction torque were minor, (2) knee extensor muscle strength (1RM, maximal voluntary concentric and isometric contraction torque) and some of the functional physical fitness tests (i.e., 30-s chair stand, 8-foot up-and-go, one leg stance) improved greater for eccentric training group than concentric training group (Figure 1), and (3) all markers of insulin sensitivity and blood lipid profiles showed improvement after eccentric training, but this was limited after concentric training (Figure 2). These support the hypotheses, and suggest that it is better to focus on eccentric than concentric contractions in resistance exercises of elderly individuals. It should be noted that the frequency of the training was only once a week, and the significant improvement of the outcome measures was achieved after only 12 training sessions in which the intensity and volume were not necessary high especially for the first 4 weeks.

### Muscle damage

The first eccentric training or concentric training session in which low-intensity eccentric (10% of 1RM) or concentric (50% of 1RM) contractions were performed, respectively, did not result in large changes in maximal voluntary concentric contraction torque, visual analog scale of muscle soreness, and plasma creatine kinase activity. Previous studies also showed that low-intensity eccentric contractions did not induce any symptoms of muscle damage (Chen et al., 2012, 2013), and muscle damage was not induced by high-intensity concentric contractions (Overend et al., 2000). Even after the 11th and 12th sessions in which maximal load based on 1RM was used, no indication of muscle damage was observed after eccentric training. This was probably due to the protective effect conferred by progressive overload started from very low-intensity, by which the knee extensors became resilient to eccentric contraction-induced muscle damage (Clarkson et al., 1992; Howatson and van Someren, 2008; Hyldahl and Hubal, 2014; LaStayo et al., 2014). It should be noted that none of the participants experienced DOMS after any of the training sessions. This might be a reason why all participants completed all 12 sessions.

The present study is the first to show that eccentric exercise training using a resistance training machine can be introduced to elderly people without muscle damage by progressively increasing the intensity and volume. It is important to note that the intensity was based on the 1RM concentric strength, and the load was 100% of 1RM for both groups at weeks 11 and 12. This was thought to be challenging especially for the concentric training group, but all participants were able to complete 6 sets of 10 contractions. This may be due to the effect of the progressive overload protocol, and the long interval between contractions (10 s) and between sets (3 min) seems to contribute to it. Generally speaking, eccentric 1RM is greater than concentric 1RM (Hortobágyi et al., 1996; Ploutz-Snyder et al., 2001; Roig et al., 2009; Nogueira et al., 2013; Douglas et al., 2016). Thus it is possible to increase the intensity further for the eccentric exercise training, and to impose greater stimulus to the muscles by increasing the intensity and volume (e.g., number of sets). It seems unlikely that severe muscle damage is induced by further increasing the intensity and volume, because of the protective effects already conferred from the previous training sessions. Thus, it is interesting to investigate whether greater effects of eccentric training on health and fitness parameters are observed, if the intensity is further increased based on eccentric strength.

### Muscle function and functional physical fitness

Eccentric training increased 1RM by 49%, maximal voluntary concentric contraction torque by 18% and maximal voluntary isometric contraction torque by 31%, whereas the respective values for concentric training were 35, 10, and 19% (Figure 1). It is important to note that the magnitude of the increase in concentric strength (i.e., 1RM, maximal voluntary concentric contraction torque) was greater for eccentric training group than concentric training group, which does not necessarily follow the training principal of specificity. The greater increases in muscle strength after eccentric training group than concentric training group are consistent with the findings of previous studies (Hortobágyi et al., 1996; Vikne et al., 2006; Marcus et al., 2008; Paschalis et al., 2011) in which sedentary young women, middle-aged men and women with type 2 diabetes mellitus, and sedentary young women were used, respectively. It is possible that the greater gain in muscle strength after eccentric training than concentric training was partially attributed to the greater muscle hypertrophy after eccentric training than concentric training. Unfortunately, the present study did not assess muscle cross sectional area or muscle fiber cross sectional area changes, although the thigh circumference showed a greater increase after eccentric training than concentric training. Previous studies (LaStayo et al., 2003, 2014; Mueller et al., 2009; Roig et al., 2009; Gault and Willems, 2013) showed greater muscle hypertrophy after eccentric than concentric training in elderly individuals. It should be noted that the 1RM test was performed before each training session, thus eccentric training group also performed submaximal and maximal concentric contractions of each leg over 12 weeks, and the total number of concentric contractions was 36–60 for each leg. This might have contributed to the training effects, but this was also performed by concentric training group. Thus, the greater increases in muscle function for eccentric training group than concentric training group were most likely due to the eccentric contractions performed by eccentric training group. This suggests that eccentric training is more effective than concentric training to attenuate sarcopenia and dynapenia, which supports the statement of Hoppeler (2016) in his recent review paper that eccentric exercise training is effective for prevention and reduction of sarcopenia.

In addition to the greater improvement of muscle strength after eccentric training than concentric training, greater improvement of 30-s chair stand, 8-foot up-and-go and one-leg stand test was observed after eccentric training than concentric training (Figure 1). It should be noted that some of the functional physical fitness tests presented a large variability between days as reported in previous studies (Rikli and Jones, 1999; Nelson et al., 2004; Lark and Pasupuleti, 2009; Fang et al., 2015; Lee et al., 2015), thus this needs to be taken into account for the interpretation of the results. However, it appears that the comparison between eccentric training and concentric training groups is still valid. It is not surprising that 30-s chair stand and 8-foot up-and-go increased more for eccentric training than concentric training group, because of the greater increase in knee extensors strength for eccentric training group than concentric training group (Figure 1). However, it is noteworthy that balance ability assessed by the one-leg stand test increased more after eccentric training than concentric training. It is possible that motor control is improved better by eccentric training than concentric training. Considering the increased risk of falling with aging, improving balance ability is important for older adults (LaStayo et al., 2003, 2014; Roig et al., 2009; Gault and Willems, 2013). It should be also noted that walking ability improved similarly between eccentric training and concentric training groups, suggesting that improving skeletal muscle function is the key to improve aerobic exercise ability (LaStayo et al., 2003, 2014; Marcus et al., 2008; Roig et al., 2009; Gault and Willems, 2013).

### Insulin sensitivity and blood lipid profiles

The effects of eccentric training on insulin sensitivity and blood lipid profiles are striking such that all measures showed significantly greater improvement after eccentric training than concentric training, and the improvement of insulin sensitivity was found only for eccentric training group (Figure 2). A previous study reported that an 8-week of knee extensors eccentric training but not concentric training decreased resting glucose and insulin concentration, HOMA and HbA1C in young women (Paschalis et al., 2011). The present study was the first to show that this was also the case for elderly men using a resistance training machine, and both studies suggest that eccentric exercise training is more effective than concentric exercise training to improve insulin sensitivity. It is important to note that muscle damage has been shown to negatively affect insulin sensitivity (Asp et al., 1996), but no indication of muscle damage was observed after any training sessions. It is not known why only eccentric exercise training improved insulin sensitivity. More studies are needed to explore the underpinning mechanisms.

Regarding blood lipid profiles, the present study showed that the decreases in TG, TC, and LDLC were greater, and the increase in HDLC was also greater after eccentric training than concentric training (Figure 2). Paschalis et al. (2011) showed that bilateral knee extensor eccentric training performed once a week for 8 weeks improved resting blood lipid profiles (TG: −15%, TC: −12%, HDLC: +11%, LDLC: −24%) in young women. It is possible that eccentric contractions increase fatty acid oxidation as shown by Peñailillo et al. (2014). Paschalis et al. (2011) speculated that the reduction in serum TC and LDLC after eccentric resistance training was due to the outflow of cholesterol from plasma into muscle, providing substrate for the synthesis of new cell membranes. It should be noted that HDLC increased only after eccentric training. It seems possible that eccentric training increased lipoprotein lipase activity in the adipose tissue and adipose tissue capillaries to better regulate lipid metabolism. Future studies are needed to investigate mechanisms underpinning how eccentric training improved blood lipid profiles better than concentric training.

### Efficacy of eccentric exercise training

The eccentric exercise used in the present study is relatively easy to perform, but a knee extension machine is required. It is interesting to investigate whether other types of eccentric exercises such as downhill walking and stair descending that people can perform without special equipment could also induce similar effects to those found in the present study. It should be also investigated if the number of resistance exercises is increased such that not only knee extensors exercise but also other exercises (e.g., arm, shoulder, trunk exercises) are included, any greater effects on functional physical fitness, insulin sensitivity and blood lipid profiles will be produced. In the present study, there was no group of participants who performed both concentric and eccentric contractions. Generally, to perform eccentric contractions, concentric contractions are also required. It is interesting to investigate whether a combination of eccentric and concentric contractions in resistance exercise training is better than performing eccentric contractions without concentric contractions with load. In the present study, a control group without any training sessions was not included, but it is assumed that changes in the outcome parameters would have been small, if any. More research is warranted to establish more effective exercise prescription, but it can be said from the present study that focusing on eccentric contractions in exercises is important.

The present study only examined healthy elderly men for their responses to the knee extensor resistance training. It is necessary to replicate this study using female participants. Potentially, the advantages of eccentric exercise training can be applied to clinical populations. Mitchell et al. (2017) have recently stated that the strong anabolic stimulation produced by eccentric exercise training benefits patients with chronic obstructive pulmonary disease, heart failure, coronary artery disease, and type 2 diabetes, as well as survivors of breast, prostate and colon cancer, and neurological conditions including Parkinson's disease, multiple sclerosis and stroke, whilst not exceeding the compromised cardiorespiratory capacity of these patients. Further, studies are warranted to investigate if the eccentric exercise protocol used in the present study and other types of eccentric exercise can be applied effectively to these clinical populations.

To summarize, the present study showed that eccentric exercise training was more effective than concentric exercise training to improve health and fitness of elderly individuals, when the eccentric exercise intensity was carefully and progressively increased not to induce any muscle damage symptoms. It is noteworthy that the exercise training was performed only once a week, and the exercise time was 30 (3 sets)–60 min (6 sets) a session. It appears that the magnitude of the eccentric exercise training effects on insulin sensitivity and blood lipid profiles are much greater than those normally found in pharmacological interventions that could cost more. Thus, it can be stated that eccentric training of the knee extensors is very cost-effective. It has been shown by previous studies that conventional resistance training is beneficial for elderly population to minimize and prevent sarcopenia that could lead to chronic diseases (Booth et al., 2012; Pedersen and Saltin, 2015; Ciolac and Rodrigues-da-Silva, 2016). It is well documented that exercise is medicine and many chronic diseases can be prevented and treated by exercises (Pedersen and Saltin, 2015). However, it has not been well recognized that eccentric exercise is more effective than concentric exercise for many aspects associated with health and fitness, thus eccentric exercise could be more effective medicine. The present study showed that eccentric contractions in resistance training should be focused more to maximize its benefits. Further, studies are necessary to investigate the mechanisms underpinning the superiority of eccentric contractions to concentric contractions for the improvement of muscle function, functional physical fitness, insulin sensitivity and blood lipid profiles.

## Author contributions

All authors contributed to the data analysis and interpretation of the data, drafting and revising the manuscript, and approved the final version of the manuscript. The original study design was made by TC and KN, and discussed with the other authors. TC, WT, GH and HC performed the data collection.

## Funding

This research was supported by the Ministry of Science and Technology (99-2410-H-415-037-MY2, 105-2410-H-003-052-MY3) and “Aim for the Top University Project” of National Taiwan Normal University (NTNU), sponsored by the Ministry of Education, TAIWAN, R.O.C. (105J2A0801).

### Conflict of interest statement

The authors declare that the research was conducted in the absence of any commercial or financial relationships that could be construed as a potential conflict of interest. The reviewer MF and handling Editor declared their shared affiliation, and the handling Editor states that the process nevertheless met the standards of a fair and objective review.

## References

[B1] Asp S. Daugaard J. R. Kristiansen S. Kiens B. Richter E. A. (1996). Eccentric exercise decreases maximal insulin action in humans: muscle and systemic effects. J. Physiol. 494, 891–898. 10.1113/jphysiol.1996.sp021541 8865083 PMC1160686

[B2] Ben-Sira D. Ayalon A. Tavi M. (1995). The effect of different types of strength training on concentric strength in women. J. Strength Cond. Res. 9, 143–148. 10.1519/00124278-199508000-00004

[B3] Blazevich A. J. Cannavan D. Coleman D. R. Horne S. (2007). Influence of concentric and eccentric resistance training on architectural adaptation in human quadriceps muscles. J. Appl. Physiol. 103, 1565–1575. 10.1152/japplphysiol.00578.2007 17717119

[B4] Booth F. W. Roberts C. K. Laye M. J. (2012). Lack of exercise is a major cause of chronic diseases. Compr. Physiol. 2, 1143–1211. 10.1002/cphy.c110025 23798298 PMC4241367

[B5] Borg G. (1970). Perceived exertion as an indicator of somatic stress. Scand. J. Rehabil. Med. 2, 92–98. 5523831

[B6] Chen H. L. Nosaka K. Chen T. C. (2012). Muscle damage protection by low-intensity eccentric contractions remains for 2 weeks but not 3 weeks. Eur. J. Appl. Physiol. 112, 555–565. 10.1007/s00421-011-1999-8 21611825

[B7] Chen H. Y. Wang H. S. Tung K. Chao H. H. (2015). Effects of gender difference and caffeine supplementation on anaerobic muscle performance. Int. J. Sports Med. 36, 974–978. 10.1055/s-0035-1550048 26252548

[B8] Chen T. C. Tseng W. C. Huang G. L. Chen H. L. Tseng K. W. Nosaka K. (2013). Low-intensity eccentric contractions attenuate muscle damage induced by subsequent maximal eccentric exercise of the knee extensors in the elderly. Eur. J. Appl. Physiol. 113, 1005–1015. 10.1007/s00421-012-2517-3 23064871

[B9] Ciolac E. G. Rodrigues-da-Silva J. M. (2016). Resistance training as a tool for preventing and treating musculoskeletal disorders. Sports Med. 46, 1239–1248. 10.1007/s40279-016-0507-z 26914266

[B10] Clarkson P. M. Nosaka K. Braun B. (1992). Muscle function after exercise-induced muscle damage and rapid adaptation. Med. Sci. Sports Exerc. 24, 512–520. 10.1249/00005768-199205000-00004 1569847

[B11] Douglas J. Pearson S. Ross A. McGuigan M. (2016). Chronic adaptations to eccentric training: a systematic review. Sports Med. [Epub ahead of print]. 10.1007/s40279-016-0628-4 27647157

[B12] Elmer S. Hahn S. McAllister P. Leong C. Martin J. (2012). Improvements in multi- joint leg function following chronic eccentric exercise. Scand. J. Med. Sci. Sports. 22, 653–661. 10.1111/j.1600-0838.2011.01291.x 21410545

[B13] Fang I. Y. Chang S. H. Ho H. H. (2015). Effects of multi-component exercise training on functional fitness in community-dwelling older adults. Phys. Educ. J. 48, 59–72. 10.3966/102472972015034801005

[B14] Franchi M. V. Atherton P. J. Reeves N. D. Flück M. Williams J. Mitchell W. K.. (2014). Architectural, functional and molecular responses to concentric and eccentric loading in human skeletal muscle. Acta Physiol. 210, 642–654. 10.1111/apha.12225 24387247

[B15] Franchi M. V. Wilkinson D. J. Quinlan J. I. Mitchell W. K. Lund J. N. Williams J. P.. (2015). Early structural remodeling and deuterium oxide-derived protein metabolic responses to eccentric and concentric loading in human skeletal muscle. Physiol. Rep. 3:e12593. 10.14814/phy2.12593 26564061 PMC4673627

[B16] Gault M. L. Willems M. E. (2013). Aging, functional capacity and eccentric exercise training. Aging Dis. 4, 351–363. 10.14336/AD.2013.0400351 24307968 PMC3843652

[B17] Gómez-Cabello A. Ara I. Gonzalez-Aguero A. Casajus J. A. Vicente-Rodriguez G. (2012). Effects of training on bone mass in older adults: a systematic review. Sports Med. 42, 301–325. 10.2165/11597670-000000000-00000 22376192

[B18] Green M. S. Doyle J. A. Ingalls C. P. Benardot D. Rupp J. C. Corana B. T. (2010). Adaptation of insulin-resistance indicators to a repeated bout of eccentric exercise in human skeletal muscle. Int. J. Sport Nutr. Exerc. Metab. 20, 181–190. 10.1123/ijsnem.20.3.181 20601735

[B19] Hawes M. R. Martin A. D. (2001). Human body composition, in Kinanthropometry and Exercise Physiology Laboratory Manual: Tests, Procedures and Data, eds Eston R. Reilly T. (London: Routledge), 42–43.

[B20] Hody S. Rogister B. Leprince P. Laglaine T. Croisier J. L. (2013). The susceptibility of the knee extensors to eccentric exercise-induced muscle damage is not affected by leg dominance but by exercise order. Clin. Physiol. Funct. Imaging 33, 373–380. 10.1111/cpf.12040 23701247

[B21] Hoppeler H. (2016). Moderate load eccentric exercise: a distinct novel training modality. *Front*. Physiol. 7:483 10.3389/fphys.2016.00483 PMC5110564 27899894

[B22] Hortobágyi T. Hill J. P. Houmard J. A. Fraser D. D. Lambert N. J. Israel R. G. (1996). Adaptive responses to muscle lengthening and shortening in humans. *J. Appl*. Physiol. 80, 765–772. 10.1152/jappl.1996.80.3.765 8964735

[B23] Howatson G. van Someren K. A. (2008). The prevention and treatment of exercise-Induced muscle damage. Sports Med. 38, 483–503. 10.2165/00007256-200838060-00004 18489195

[B24] Huang C. C. Tsai C. L. Wang H. S. Ding S. T. Lin H. F. (2015). Effects of different types of eccentric exercise on pulse wave velocity and adiponectin response. *Phys. Educ*. J. 48, 19–32. 10.3966/102472972015034801002

[B25] Hyldahl R. D. Hubal M. J. (2014). Lengthening our perspective: morphological, cellular, and molecular responses to eccentric exercise. Muscle Nerve 49, 155–170. 10.1002/mus.24077 24030935

[B26] Jamurtas A. Z. Garyfallopoulou A. Theodorou A. A. Zalavras A. Paschalis V. Deli C. K.. (2013). A single bout of downhill running transiently increases HOMA-IR without altering adipokine response in healthy adult women. Eur. J. Appl. Physiol. 113, 2925–2932. 10.1007/s00421-013-2717-5 24068487

[B27] Lark S. D. Pasupuleti S. (2009). Validity of a functional dynamic walking test for the elderly. Arch. Phys. Med. Rehabil. 90, 470–474. 10.1016/j.apmr.2008.08.221 19254613

[B28] LaStayo P. C. Ewy G. A. Pierotti D. D. Johns R. K. Lindstedt S. (2003). The positive effects of negative work: increased muscle strength and decreased fall risk in a frail elderly population. J. Gerontol. A Biol. Med. Sci. 58, M419–M424. 10.1093/gerona/58.5.m419 12730250

[B29] LaStayo P. Marcus R. Dibble L. Frajacomo F. Lindstedt S. (2014). Eccentric exercise in rehabilitation: safety, feasibility, and application. J. Appl. Physiol. (1985). 116, 1426–1434. 10.1152/japplphysiol.00008.2013 23823152

[B30] Lee Y. S. Chang W. H. Lin T. C. Shiang T. Y. (2015). Does functional fitness decline in accordance with our expectation? – a pilot study in healthy female. *BMC Sports Sci. Med*. Rehabil. 7:17 10.1186/s13102-015-0012-y PMC4498516 26167287

[B31] Marcus R. L. Smith S. Morrell G. Addison O. Dibble L. E. Wahoff-Stice D.. (2008). Comparison of combined aerobic and high-force eccentric resistance exercise with aerobic exercise only for people with type 2 diabetes mellitus. Phys. Ther. 88, 1345–1354. 10.2522/ptj.20080124 18801851 PMC2579905

[B32] Mitchell W. K. Taivassalo T. Narici M. V. Franchi M. V. (2017). Eccentric exercise and the critically Ill patient. *Front*. Physiol. 8:120 10.3389/fphys.2017.00120 PMC5330179 28293200

[B33] Mueller M. Breil F. A. Vog M. Steiner R. Lippuner K. Popp A.. (2009). Different response to eccentric and concentric training in older men and women. Eur. J. Appl. Physiol. 107, 145–153. 10.1007/s00421-009-1108-4 19543908

[B34] Nelson M. E. Layne J. E. Bernstein M. J. Nuernberger A. Castaneda C. Kaliton D.. (2004). The effects of multidimensional home-based exercise on functional performance in elderly people. J. Gerontol. A Biol. Sci. Med. Sci. 59, 154–160. 10.1093/gerona/59.2.M154 14999030

[B35] Nickols-Richardson S. M. Miller L. E. Wootten D. F. Ramp W. K. Herbert W. G. (2007). Concentric and eccentric isokinetic resistance training similarly increases muscular strength, fat-free soft tissue mass, and specific bone mineral measurements in young women. Osteoporos. Int. 18, 789–796. 10.1007/s00198-006-0305-9 17264975

[B36] Nogueira F. R. Libardi C. A. Vechin F. C. Lixandrao M. E. de Barros Berton R. P. de Souza T. M.. (2013). Comparison of maximal muscle strength of elbow flexors and knee extensors between younger and older men with the same level of daily activity. Clin. Interv. Aging 8, 401–407. 10.2147/CIA.S41838 23610518 PMC3629865

[B37] Overend T. J. Versteegh T. H. Thompson E. Birmingham T. B. Vandervoort A. A. (2000). Cardiovascular stress associated with concentric and eccentric isokinetic exercise in young and older adults. J. Gerontol. A Biol. Sci. Med. Sci. 55, B177–B182. 10.1093/gerona/55.4.b177 10811144

[B38] Paschalis V. Nikolaidis M. G. Theodorou A. A. Panayiotou G. Fatouros I. G. Koutedakis Y.. (2011). A weekly bout of eccentric exercise is sufficient to induce health-promoting effects. Med. Sci. Sports Exerc. 43, 64–73. 10.1249/MSS.0b013e3181e91d90 20508540

[B39] Peñailillo L. Blazevich A. Nosaka K. (2014). Energy expenditure and substrate oxidation during and after eccentric cycling. Eur. J. Appl. Physiol. 114, 805–814. 10.1007/s00421-013-2816-3 24390692

[B40] Pedersen B. K. Saltin B. (2015). Exercise as medicine - evidence for prescribing exercise as therapy in 26 different chronic diseases. Scand. J. Med. Sci. Sports 25, 1–72. 10.1111/sms.12581 26606383

[B41] Ploutz-Snyder L. L. Giamis E. L. Formikell M. Rosenbaum A. E. (2001). Resistance training reduces susceptibility to eccentric exercise-induced muscle dysfunction in older women. J. Gerontol. A Biol. Med. Sci. 56, B384–B390. 10.1093/gerona/56.9.b384 11524439

[B42] Rikli R. E. Jones C. J. (1999). Functional fitness normative scores for community- residing older adults, ages 60-94. J. Aging Phys. Act. 7, 162–181. 10.1123/japa.7.2.162

[B43] Roig M. O'Brien K. Kirk G. Murray R. McKinnon P. Shadgan B.. (2009). The effects of eccentric versus concentric resistance training on muscle strength and mass in healthy adults: a systematic review with meta- analysis. Br. J. Sports Med. 43, 556–568. 10.1136/bjsm.2008.051417 18981046

[B44] Tracy B. L. Enoka R. M. (2002). Older adults are less steady during submaximal isometric contractions with the knee extensor muscles. J. Appl. Physiol. (1985). 92, 1004–1012. 10.1152/japplphysiol.00954.2001 11842033

[B45] Vikne H. Refsnes P. E. Ekmark M. Medbø J. I. Gundersen V. Gundersen K. (2006). Muscular performance after concentric and eccentric exercise in trained men. Med. Sci. Sports Exerc. 38, 1770–1781. 10.1249/01.mss.0000229568.17284.ab 17019299

[B46] Vogt M. Hoppeler H. H. (2014). Eccentric exercise: mechanisms and effects when used as training regime or training adjunct. J. Appl. Physiol. (1985). 116, 1446–1454. 10.1152/japplphysiol.00146.2013 24505103

[B47] Wang H. H. Chen W. H. Liu C. Yang W. W. Huang M. Y. Shiang T. Y. (2014). Whole-body vibration combined with extra-load training for enhancing the strength and speed of track and field athletes. *J. Strength Cond*. Res. 28, 2470–2477. 10.1519/JSC.0000000000000437 24662223

[B48] Zeppetzauer M. Drexel H. Vonbank A. Rein P. Aczel S. Saely C. H. (2013). Eccentric endurance exercise economically improves metabolic and inflammatory risk factors. Eur. J. Prevent. Cardiol. 20, 577–584. 10.1177/2047487312444236 22505055

